# Supplementary material for: Identification of photoperiod-induced specific miRNAs in the adrenal glands of Sunite sheep (Ovis aries)
Source: Front Vet Sci. 2022 Jul 22;9:888207. doi: 10.3389/fvets.2022.888207 (PMC9354845; doi:10.3389/fvets.2022.888207)
Supplement: Supplementary file 1 [file Data_Sheet_1.ZIP › Supplementary file/miRDeep2 file.zip!/miRDeep2 file/Know_Structure/LP42A1/oar-mir-377.pdf]

miRBase precursor : oar-mir-377
Total read count : 55
oar-miR-377-5p read count : 17
oar-miR-377-3p read count : 30
remaining reads : 8

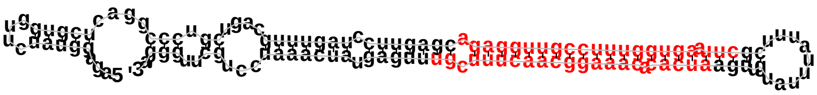

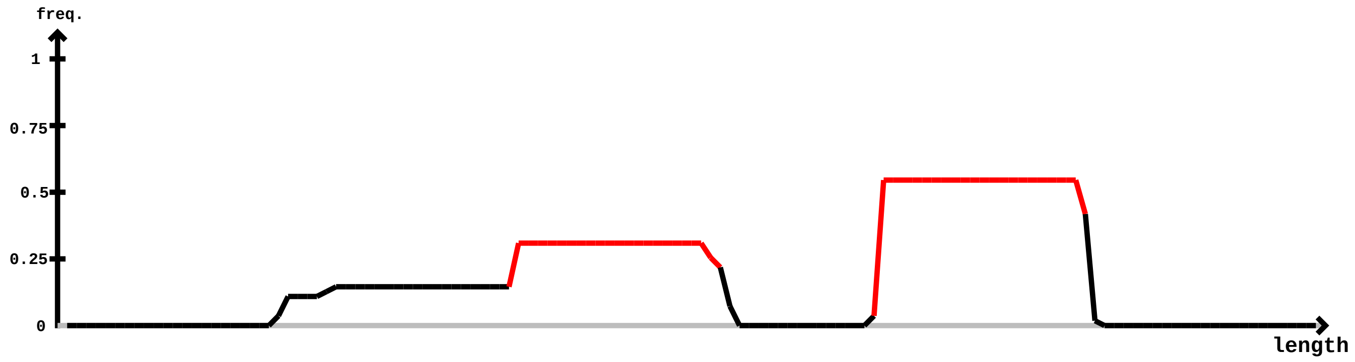


```
                                                                                   oar-miR-377-3p
                                             oar-miR-377-5p
5'- agagguaucuuggugcucaggcccugcugacguuugauccuugagcagagguugccuuuggugaauucgcuuuauuuauguugaaucacacaaaggcaacuuucguuugaguaucaaauccugcuuggga   -3'   exp
    ...(((((....)))))....(((.((....(((((((.(((((((.(((((((((((((((((.(((((.........).)))))))).))))))))))))).))))))).)))))))...))..))).    reads   mm
    .....................cccugcugacguuugauccuugagc....................................................................................    2       0
    ......................ccugcugacguuugauccuugagc....................................................................................    4       0
    ..........................cugacguuugauccuugagc....................................................................................    1       0
    ...........................ugacguuugauccuugagc....................................................................................    1       0
    ..............................................agagguugccuuuggugaau................................................................    3       0
    ..............................................agagguugccuuuggugaauu...............................................................    2       0
    ..............................................agagguugccuuuggugaauuc..............................................................    8       0
    ..............................................agagguugccuuuggugaauucg.............................................................    4       0
    ...................................................................................aaucacacaaaggcaacuuucgu........................    2       0
    ....................................................................................aucacacaaaggcaacuuucg.........................    7       0
    ....................................................................................aucacacaaaggcaacuuucgu........................    20      0
    ....................................................................................aucacacaaaggcaacuuucguu.......................    1       0
```
